# Transcriptome analysis reveals the regulation of brassinosteroids on petal growth in *Gerbera hybrida*

**DOI:** 10.7717/peerj.3382

**Published:** 2017-05-31

**Authors:** Gan Huang, Meixiang Han, Wei Yao, Yaqin Wang

**Affiliations:** Guangdong Provincial Key Laboratory of Biotechnology for Plant Development, School of Life Sciences, South China Normal University, Guangzhou, China

**Keywords:** Brassinolide, Regulation, *Gerbera hybrida*, Petal growth, RNA-seq

## Abstract

*Gerbera hybrida* is a cut-flower crop of global importance, and an understanding of the mechanisms underlying petal development is vital for the continued commercial development of this plant species. Brassinosteroids (BRs), a class of phytohormones, are known to play a major role in cell expansion, but their effect on petal growth in *G. hybrida* is largely unexplored. In this study, we found that the brassinolide (BL), the most active BR, promotes petal growth by lengthening cells in the middle and basal regions of petals, and that this effect on petal growth was greater than that of gibberellin (GA). The RNA-seq (high-throughput cDNA sequencing) technique was employed to investigate the regulatory mechanisms by which BRs control petal growth. A global transcriptome analysis of the response to BRs in petals was conducted and target genes regulated by BR were identified. These differentially expressed genes (DEGs) include various transcription factors (TFs) that were activated during the early stage (0.5 h) of BL treatment, as well as cell wall proteins whose expression was regulated at a late stage (10 h). BR-responsive DEGs are involved in multiple plant hormone signal pathways, hormone biosynthesis and biotic and abiotic stress responses, showing that the regulation of petal growth by BRs is a complex network of processes. Thus, our study provides new insights at the transcriptional level into the molecular mechanisms of BR regulation of petal growth in *G. hybrida*.

## Introduction

Brassinosteroids (BRs) are plant-specific steroid hormones involved in many growth and developmental processes, such as cell division and elongation, leaf expansion, vascular differentiation, stress tolerance, senescence and stomatal development (Bajguz & Hayat, 2009; Clouse & Sasse, 1998; Gudesblat et al., 2012; Kim et al., 2012). For example, the role of BR in cell division has been shown in the model plant *Arabidopsis thaliana* by analysis of mutants defective in BR biosynthesis, such as *dwf7-1*, which exhibits a slow cell division rate, and *det2*, in which transcription of the gene encoding cyclin D3 (a protein regulating G1/S transition in the cell cycle) increases after treatment with the 2,4-epibrassinolide (Cheon et al., 2010; Hu, Bao & Li, 2000). Similarly, longitudinal cell expansion is greatly reduced in BR mutants such as *cbb, dwf4, cpd* and *dim,* indicating a role for BRs in cell expansion *(Azpiroz & Feldmann, 1998; Szekeres et al., 1996; Takahashi et al., 1995)*.

Coordination of organ growth with cell division, and of cell expansion with cell differentiation, is essential for the determination of organ size in plants (Mizukami, 2001). Large-scale analysis using microarray or proteomic techniques has also demonstrated the importance of BR-regulated genes in cell expansion. These genes are involved in cell wall modification, cellulose biosynthesis, ion and water transport, and cytoskeleton rearrangement (Clouse & Sasse, 1998; Kim & Wang, 2010; Vert et al., 2005). For example, DEVELOPMENTALLY REGULATED PLASMA MEMBRANE POLYPEPTIDE (DREPP), which was first identified as a BR-regulated protein in proteomic studies (Tang et al., 2008), can enhance cell elongation in a BR-deficient mutant, indicating that DREPP is a positive regulator of the BR response (Sun et al., 2010). Further studies in Arabidopsis showed that *BRASSINAZOLE-RESISTANT1 (BZR1)* and *BRI1-EMS-SUPPRESSOR1 (BES1)* encode two major transcription factors (TFs) that regulate cell division and elongation through a large number of BR -responsive genes (Guo et al., 2009; He et al., 2005; Sun et al., 2010; Wang et al., 2002; Xie, Yang & Wang, 2011; Yin et al., 2002; Yu et al., 2011).

*Gerbera hybrida* is a member of the sunflower family (Asteraceae), one of the largest clades of angiosperms. The inflorescence of *G. hybrida*, which consists of three types of flowers with ray, trans or disc petals, has been used as a model species to study developmental processes during organogenesis in the Asteraceae (Kotilainen et al., 1999; Yu et al., 1999). Consequently, in recent decades, many studies have been conducted on the molecular regulation of flower type differentiation and inflorescence development in *G. hybrida*. For instance, the CYC2 subclade of CYC/TB1-like TCP domain TFs has been shown to regulate flower-type identity in Asteraceae. Thus, *GhCYC2*, *GhCYC3* and *GhCYC4* control the differentiation and petal growth of ray flowers by modulating cell proliferation until the final size and shape of the petals is achieved (Broholm & Elomaa, 2008; Chapman et al., 2012; Fambrini, Salvini & Pugliesi, 2011; Kim et al., 2008; Tähtiharju et al., 2012). Another such TF, *GhCYC5*, is probably involved in promoting the flower initiation rate during expansion of the capitulum (Juntheikki-Palovaara et al., 2014). Research on orthologs of the flower meristem identity genes *LEAFY (LFY)* and *UNUSUAL FLORAL ORGANS (UFO)* in *G. hybrida* showed that *GhUFO* is the major regulator of flower meristem identity, while *GhLFY* has developed a novel, homeotic function during the evolution of head-like inflorescences (Zhao et al., 2016).

In our previous studies, we found that ray petals exhibit substantial cell expansion only after developmental stage 3 (Meng & Wang, 2004). Further work demonstrated that the basal region of ray petals was most sensitive to treatment with gibberellins (GAs) and abscisic acid (ABA) and that these phytohormones regulated cell expansion in an antagonistic manner (Li et al., 2015). Cell elongation is regulated not only by GA, but also by BR in Arabidopsis (Gallego-Bartolomé & Blázquez, 2012; Li et al., 2012). However, little is known about the role and regulatory mechanisms of BR in *G. hybrida* petal growth. In this study, we used the RNA-seq technique to perform global transcriptomic profiling in petals of *G*. *hybrida* and to identify differentially expressed genes (DEGs) responsive to BR treatment. The results showed that many DEGs are involved not only in cell wall organization and transcriptional regulation, but also in multiple plant hormone signaling pathways, hormone biosynthesis, and biotic and abiotic stress responses, indicating that the regulatory effect of BR on petal growth is a complex network of processes.

## Materials and Methods

### Plant material and growth conditions

*G. hybrida* “Shenzhen No. 5” seedlings were grown in a greenhouse at Zengcheng Ornamental Center (Guangzhou, China) as described by Zhang et al. (2012) at a temperature of 26/18 °C (day/night) and relative humidity of 65–80%. The developmental stages of inflorescence were defined according to Meng & Wang (2004). Inflorescences at stage 2, with a ray petal length of about 8 mm, were used for the *in vivo* experiments, while ray petals at stage 3 were used for *in vitro* studies.

### Hormone and inhibitor treatments *in vivo* and *in vitro*

Five to six inflorescences at stage 2 were selected for *in vivo* treatment. The inflorescences were treated by spraying with 3–5 ml 10 µM brassinolide (BL), 10 µM GA_3_, 10 µM BRZ (brassinazole, a widely used inhibitor of BL biosynthesis), BL + GA_3_, or BL + BRZ once a day, using 0.1% ethanol as a control. The inflorescences were sampled after nine days. Ten petals of the outermost whorl of ray flowers were detached from the inflorescences at stage 3 for *in vitro* treatment, following the method described previously (Huang et al., 2008; Zhang et al., 2012). Briefly, the detached petals were placed on two layers of Whatman filter paper soaked in deionized water with or without hormones and/or inhibitor, and treated for nine days. The influence of the duration of BL treatment on petal growth was evaluated by *in vitro* experiments using the detached petals treated with 10 µM BL for 0.5, 0.75, 1, 2, 4, 10, 12 and 24 h, using deionized water (ddH_2_O) as a control. Hormones and inhibitors were obtained from Sigma-Aldrich (Shanghai, China). All experiments were replicated at least three times.

### Observation and measurement of petal and cell length

To measure petal elongation *in vivo*, whole petals from more than six inflorescences were collected for each treatment. For each treatment, 60 whole petals from six inflorescences (10 each) were collected. Their images were scanned using an Epson-G850A scanner (Epson, China) and their lengths were measured using Image J software (http://rsb.info.nih.gov/ij/; NIH, MD, USA). Data were expressed as the averages of 60 petals. The lengths of three regions (top, middle and basal) of petals treated *in vitro* were measured before and after treatment. Data were expressed as the averages of ten petals for each treatment. Elongation rates were calculated according to the equation: elongation rate = (L*f*–Li)/Li × 100%, where L*f* is the petal length after treatment, and Li is the initial length before treatment.

To measure cell length in petals, a 1 mm^2^ petal block was dissected from the center of each region and stained by immersion in 0.1 mg mL^−1^ propidium iodide for 5 min at room temperature. The stained petal block was rinsed thoroughly with deionized water and flattened on a glass slide. Abaxial epidermal cell images were obtained with a laser confocal scanning microscope (LSM710/ConfoCor2; Carl-Zeiss, Jena, Germany) and their cell length was measured using Image J software. More than 100 cells of each of 10 petals from different inflorescences were randomly selected and measured. The cell elongation rate was calculated using the same equation described above. Data were analyzed using SPSS Statistics v. 18.0 (SPSS Inc., Chicago, IL, USA). Duncan’s test was applied to assess the differences between different treatments.

### RNA-seq

For each treatment, 200 petals from 20 inflorescences at stage 3 were treated with BL or ddH_2_O for 0.5 or 10 h, using untreated material as the control. After treatment, petals were frozen in liquid nitrogen and stored at −80 °C. Total RNA was extracted using the TRIzol^®^ reagent (Invitrogen, Carlsbad, CA, USA) according to the manufacturer’s instructions. DNase I (TaKaRa, Japan) was used to digest genomic DNA. The quality of total RNA was checked with an Agilent 2100 Bioanalyzer (Agilent Technologies, Palo Alto, CA, USA). Samples with an RNA integrity number >8 were used to prepare cDNA libraries, as previously described (Kuang et al., 2013). Illumina sequencing was performed at Beijing Biomarker Technologies Co. Ltd. (Beijing, China). Five sets of raw reads were obtained, corresponding to Mock (untreated), H 0.5 (0.5 h ddH_2_O treatment), B 0.5 (0.5 h BL treatment), H 10 (10 h ddH_2_O treatment), and B 10 (10 h BL treatment).

### Data processing and analysis

Raw read processing and primary bioinformatics analysis of the transcript datasets were conducted at Beijing Biomaker Technologies. In brief, clean reads were obtained from the raw data by removing adaptor sequences and reads of low-quality (*Q* value ≤ 5), fragments less than 20 bp and reads with more than 10% unknown bases. The clean reads were mapped to a *G*. *hybrida* transcriptome assembled previously (Kuang et al., 2013) using SOAPaligner/soap2 (Li et al., 2009). Mismatches of no more than two bases were allowed, with separate alignments being performed for each sample independently. Transcripts mapped by at least one read in at least one sample were identified for further analysis. Transcript abundance was expressed as RPKM (reads mapped per 1,000 bp per million sequenced reads) (Mortazavi et al., 2008). RPKM values presenting as “0” were artificially set to “0.001” for subsequent analysis. To analyze DEGs, a modified method described previously (Audic & Claverie, 1997) was used. Comparisons of RPKM between treatments (H 0.5 vs. B 0.5, H 10 vs. B 10) were performed for each transcript. Transcripts with a fold-change of ≥2 and a false discovery rate (FDR) <0.05 in at least one comparison were considered to be significantly differentially expressed and therefore to represent DEGs.

DEGs were used for BLASTX screening of protein databases, including the nr (the non-redundant protein database at NCBI), Swiss-Prot, COG, KEGG, and GO protein databases. GO annotation was performed using the Blast2GO program (Conesa et al., 2005). For GO enrichment analysis, GO annotations for each DEG were retrieved by mapping to GO terms in the database at http://www.geneontology.org. GO terms for Biological Processes (GO-BP) with a FDR ≥ 0.05 were considered significant. For KEGG pathway analysis, KEGG orthology terms for DEGs were retrieved from the KEGG pathway database (http://www.genome.jp/kegg/). The enrichment analysis was performed by comparing the observed DEG count with the expected count based on the transcriptome reported previously (Kuang et al., 2013). Hierarchical clustering analysis was performed using MeV 4.9.0 (http://www.tm4.org/) by considering the RPKM value as the normalized transcript level. Cluster analysis of gene expression patterns was performed using cluster software (Hoon et al., 2004) and Java Treeview software (Saldanha, 2004).

### Real-time PCR verification of RNA-seq data

Total RNA was extracted as described above. Each RNA sample was treated with RNase-free DNase I (TaKaRa, Shiga, Japan) following the manufacturer’s protocol to remove any residual genomic DNA. DNase I-treated RNA (2 µg) was subjected to reverse transcriptase reactions using oligo(dT) primer and PrimeScript™ Reverse Transcriptase (TaKaRa, Shiga, Japan) according to the manufacturer’s protocol. For qRT-PCR, transcripts of target genes were amplified in a 20 µl reaction containing 2 µl cDNA (corresponding to 20 ng RNA), 1 µl primers and 5 µl SoFast™ EvaGreen^®^ Supermix (Bio-Rad, Hercules, CA, USA) (Su et al., 2016). The primers are listed in Table S1. Expression levels of the tested genes were normalized to that of the *GhACTIN* (AJ763915) gene, as previously described (Kuang et al., 2013). Three biological replicates were performed per sample.

## Results

### The brassinolide modulates petal growth *in vivo* and *in vitro*

**Figure 1:**
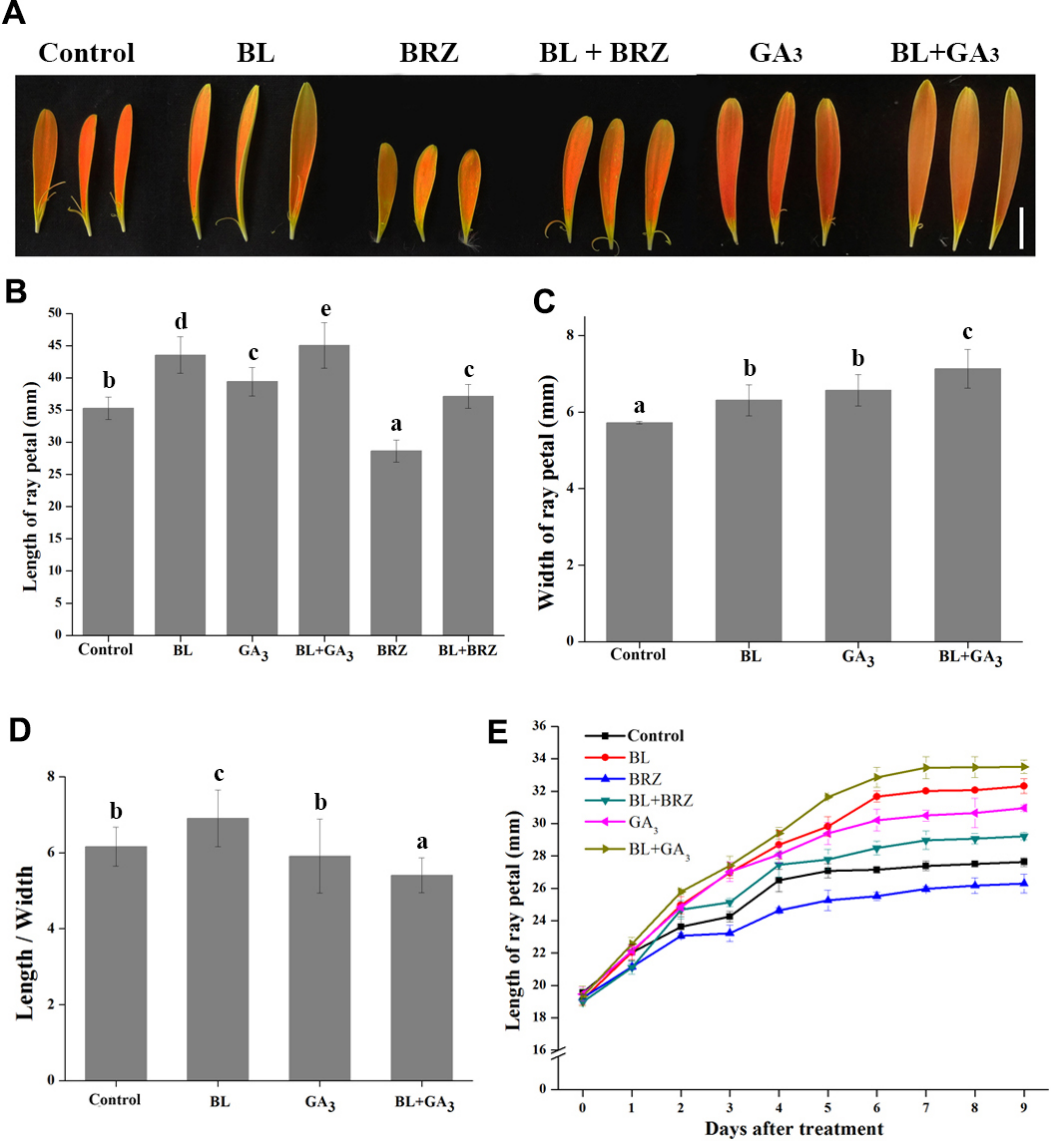
The effects of BRs and GAs on petal growth in *G. hybrida*. *G. hybrida* grew in a greenhouse under the conditions described in ‘Materials and Methods’. Plants with inflorescences at stage 2 were sprayed with deionized water (with 0.1% ethanol) (Control), 10 µM BL, 10 µM GA_3_, 10µM BRZ, a mixture of 10 µM BL and 10 µM GA_3_ (BL + GA_3_), or a mixture of 10 µM BL and 10 µM BRZ (BL + BRZ) and were subjected to morphological characterization (A), then the petal length (B), width (C) and length/width (D) were measured and calculated after nine days of treatment. Five to six inflorescences were measured for each treatment (Scale bar represents 1 cm. Value = means ± SE, *n* > 10, letters above the bars indicate significant differences between the respective values (*p* < 0.05)). (E) Time-course dynamics of petal length under different treatments. Detached petals were used in this experiment, at least 10 petals for each treatment were cultured for nine days.

To examine the role of BRs in the regulation of petal growth, both *in vivo* and *in vitro* experiments were carried out. *G. hybrida* plants treated with BL had an average petal length of 43.55 mm compared to 35.27 mm in the untreated control, whereas treatment with BRZ, a BR biosynthetic inhibitor, resulted in an average petal length of 28.64 mm. The suppression of petal elongation by BRZ could be countered by spraying exogenous BL on the petals (Figs. 1A and 1B). These data clearly show that BL stimulates petal elongation *in vivo*.

Because GAs and BRs coordinately regulate plant growth in Arabidopsis (Li et al., 2012), and GAs promotes petal elongation in *G. hybrida* (Li et al., 2015), we tested the effect of combined treatment with GA_3_ and BL on petal growth in *G. hybrida*. The results showed that the length and width of the petals were both increased after the combined treatment, with a higher elongation rate than that of either BR or GA treatment alone (Figs. 1C and 1D; *p* < 0.05).

Detached petals were used in the *in vitro* experiment. After 9 days of hormone and/or inhibitor treatment, petal elongation was greater with BL treatment than with GA treatment, which gave average petal lengths of 32.3 mm and 30.9 mm (*p* < 0.05), respectively. BRZ-mediated suppression of petal length was reversed by the application of exogenous BL. Moreover, petals treated with both BL and GA_3_ elongated significantly more than with other treatments (Fig. 1E; *p* < 0.05), suggesting that BRs and GAs can coordinately regulate petal elongation in *G*. *hybrida*. These results are consistent with those obtained from *in vivo* experiments.

### Brassinosteroids preferentially stimulate elongation of the middle and basal regions of petals

**Figure 2:**
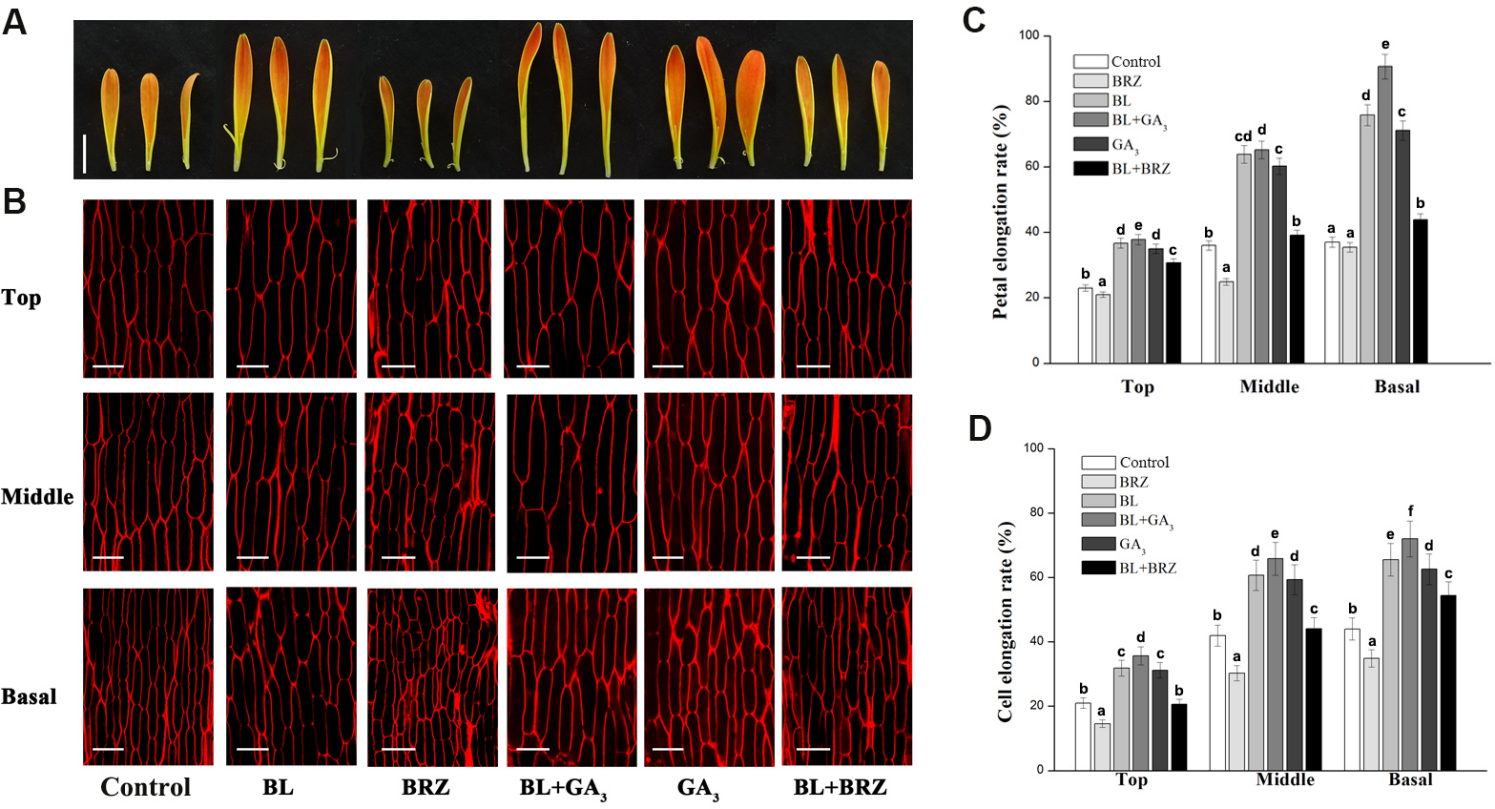
The effects of BRs and GAs on cell elongation of petals in *G. hybrida*. Petals of inflorescences at stage 3 were detached and placed on double filter papers soaked with deionized water (with 0.1% ethanol) (Control), 10 µM BL, 10 µM GA_3_, 10µM BRZ, BL + GA_3_ or BL + BRZ for nine days (A). The petals were then used for morphological characterization of adaxial epidermal cell in three different regions (top, middle and basal) using a confocal microscope (B) and measurement of elongation rate of each petal region (C), (*n* = 10) or cell (D), (*n* > 100). Three biological replicates were performed for each measurement (Scale bar represents 1 cm (A) or 50 µm (B). Value = means ± SE, letters above the bars indicate significant differences between the respective values (*p* < 0.05)).

We next asked whether different regions of the detached petals experienced different elongation rates when treated with BR (Li et al., 2015). After nine days BL treatment, the elongation rates of the top, middle and basal regions of petals were 36.7, 63.8 and 75.8%, respectively, compared to 23.0, 36.5 and 37.2% in the same regions of the untreated controls. GA treatment also promoted elongation of the three petal regions, giving rates of 35.0, 60.2 and 71.1%, respectively. Elongation rates following BRZ treatment, however, were 21.1, 24.8 and 35.4% for the three regions, respectively; these were significantly lower than those of other treatments and also controls (Figs. 2A and 2C; *p* < 0.05). Interestingly, elongation rates were extremely high in the middle and basal regions of the petals treated with BL and GA_3_ combined, indicating a cooperative effect of the two phytohormones mainly concentrated in these regions.

We then looked at the petal cells and found that cell elongation rates were greatly increased in the presence of BL, with values of 31.9, 60.7 and 65.6% in the top, middle and basal regions, compared to 21.2, 42.0 and 43.8%, respectively, in the control. In contrast, cell elongation rates were suppressed by BRZ treatment, with values of only 14.6, 30.2 and 34.8% in the three regions, respectively. The cell elongation rates in the top, middle and basal regions were 35.7, 65.8 and 72.2%, respectively, after combined BL and GA_3_ treatment, compared to 31.2, 59.3 and 62.5%, respectively, with GA_3_ treatment alone (Figs. 2B and 2D; *p* < 0.05). These results indicate that petal elongation is associated with cell elongation, and that both BRs and GAs exert their elongation effect predominantly on cells of the middle and basal regions of *G. hybrida* petals.

### Global sequencing analysis

**Figure 3:**
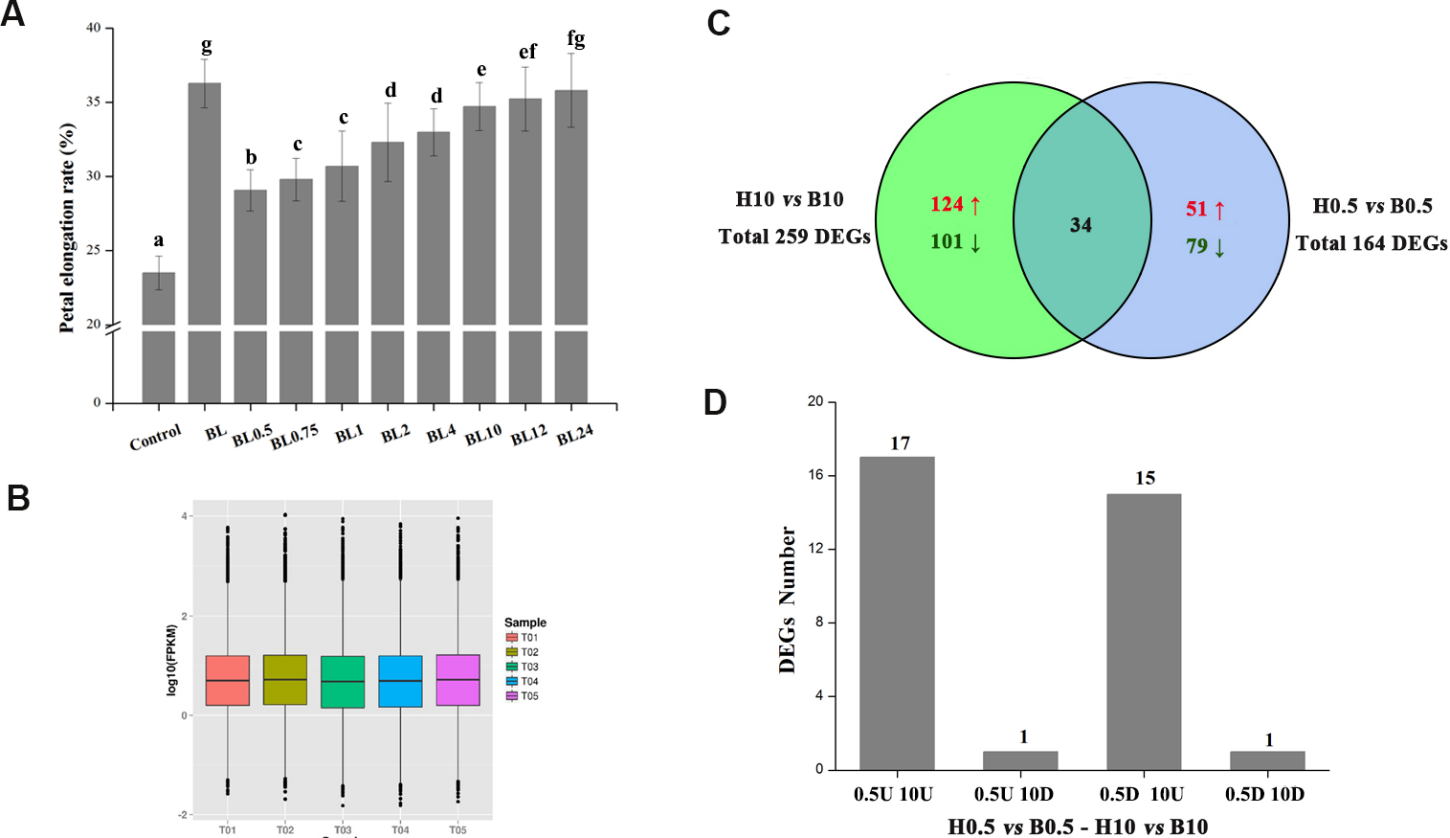
The elongation rates of petals and global analysis of transcript profiles in different treatments. (A) Detached petals at stage 3 were placed on double filter papers soaked with 10 µM BL for 0.5, 0.75, 1, 2, 4, 10, 12 and 24 h, and then transferred to papers soaked with deionized water. Petals were cultured for a total of 72 h and then the elongation rates were measured. Control: petals kept on double filter papers soaked with deionized water for 72 h. BL: petals kept on double filter papers soaked with10 µM BL for 72 h. (The experiment was repeated at least three times. Value = means ± SE, letters above the bars indicate significant differences between the respective values (*p* < 0.05)) (B) Global transcript profiles of different treatments. T01: petals without treatment (Mock), T02: H_2_O treatment for 0.5 h, T03: BL treatment for 0.5 h, T04: H_2_O treatment for 10 h, T05: BL treatment for 10 h. Each box plot shows the distribution of the relative transcription level (log_10_ (RPKM)) of genes with at least one read mapped to the transcriptome of G. *hybrida* in one sample. (C) Venn diagram showing the distribution of DEGs at 0.5 h or 10 h of BL treatment. (D) The number of DEGs in 0.5U 10U, 0.5U 10D, 0.5D 10U and 0.5D 10D. 0.5U 10U refers to DEGs up-regulated at both 0.5 h and 10 h, 0.5U 10D refers to DEGs up-regulated at 0.5 h and down-regulated at 10 h, 0.5D 10U refers to DEGs down-regulated at 0.5 h and up-regulated at 10 h, 0.5D 10D refers to DEGs down-regulated at both 0.5 h and 10 h.

To explore the influence of different BL treatments on the petals at different time points, the growth dynamics of petals were tested *in vitro*. As shown in Fig. 3A, the petals after 0.5 h BL treatment were longer than those of the control (an increase of 29.8% vs. 23.5%, *p* < 0.05). The elongation rate increased with time until the 10 h timepoint, at which it was 34.7%. After 10 h, the elongation rate changed little during the remainder of the experiment. Five treatments, including BL treatment for 0.5 or 10 h, H_2_O treatment for 0.5 or 10 h, and the petals without treatment (Mock), were selected for RNA-seq.

The BL-associated gene regulatory networks that modulate petal growth were explored using RNA-seq data. A total of 16.82 GB clean reads were generated from the five samples, which had phred-like quality scores ranging from 88.71 to 89.09% at the Q30 level, and GC contents between 45.49% and 46.01% (Table 1). All reads were then mapped onto a previously published transcriptome after removing “dirty” data, with the mapped ratios (proportion of reads mapping to the transcriptome) of the five samples ranging from 71.33% to 72.86% (Table 1). The global distribution (using the relative transcription level (log_10_ (RPKM)) of genes) of each sample is shown in Fig. 3B. Finally, we identified 164 and 259 DEGs after BL treatment for 0.5 and 10 h, respectively, of which 34 transcripts were common to both datasets (Fig. 3C). These 34 transcripts were divided into four groups based on their expression patterns (0.5U 10U: transcripts up-regulated at both 0.5 h and 10 h timepoints; 0.5U 10D: transcripts up-regulated at 0.5 h and down-regulated at 10 h; 0.5D 10U: transcripts down-regulated at 0.5 h and up-regulated at 10 h; 0.5D 10D: transcripts down-regulated at both 0.5 h and 10 h). There were 17 genes with the 0.5U 10U pattern, including genes encoding three zinc finger TFs and five heat shock proteins, plus a TF that regulates heat shock protein genes. Fifteen genes fell into the 0.5D 10U category, many of them involved in secondary metabolite biosynthesis, transport and catabolism. Only one gene each followed the 0.5U 10D and 0.5D 10D expression patterns, encoding a member of the cytochrome P450 family and a homolog of the TF WRKY70, respectively (Table 2).

**Table 1:** Summary of the mapping reads identified by RNA-seq.

Samples	Read number	Base number	Mapped reads	Mapped ratio	GC content	% ≥Q30
Mock	11,868,506	2,990,213,769	8,647,966	72.86%	45.49%	89.09%
H 0.5	11,042,298	2,781,854,022	7,876,652	71.33%	45.70%	89.07%
B 0.5	14,823,296	3,734,419,611	10,623,680	71.66%	46.01%	88.71%
H 10	14,971,100	3,771,890,604	10,741,596	71.74%	45.88%	89.05%
B 10	14,060,613	3,542,095,965	10,041,466	71.41%	45.85%	88.82%

**Table 2:** DEGs appeared in both H 0.5 *vs.* B 0.5 and H 10 *vs.* B 10.

Unigene ID	H 0.5 *vs.* B0.5	H10 *vs.* B10	Annotation
**0.5U 10U**			
GACN01031640.1	2.14	1.93	Heat stress transcription factor A-7a
GACN01005379.1	1.74	1.95	RING-H2 zinc finger
GACN01029771.1	1.74	1.47	Hsp20/alpha crystallin family
GACN01043473.1	1.95	1.28	Heat shock factor protein HSF30
GACN01001749.1	1.52	1.41	Heat shock protein 83
GACN01026124.1	1.34	1.13	PREDICTED: uncharacterized protein
GACN01046241.1	3.30	2.99	Cytokinin dehydrogenase 3
GACN01043054.1	1.85	1.24	C3HC4 type RING finger
GACN01002323.1	1.22	1.41	Hsp70 protein
GACN01029465.1	1.72	1.08	Hsp20/alpha crystallin family
GACN01022828.1	1.66	3.14	PREDICTED: uncharacterized protein
GACN01042640.1	1.94	1.76	Multiprotein-bridging factor 1c
GACN01039604.1	1.91	1.81	Two-component response regulator ARR4
GACN01006153.1	1.41	1.37	C3HC4 type (RING finger)
GACN01005141.1	1.79	1.11	Histidine kinase 4
GACN01042426.1	1.33	1.05	GEM-like protein 5
GACN01015877.1	2.65	3.65	Cytokinin dehydrogenase 1
**0.5U 10D**			
GACN01030442.1	1.51	−1.46	Secondary metabolites biosynthesis, transport and catabolism Cytochrome P450
**0.5D 10U**			
GACN01011667.1	−1.30	1.19	Delta fatty acid dehydrogenase
GACN01011938.1	−1.23	1.15	WRKY transcription factor 40
GACN01014026.1	−1.00	1.46	Secondary metabolites biosynthesis, transport and catabolism Cytochrome P450
GACN01046042.1	−1.00	1.26	Secondary metabolites biosynthesis, transport and catabolism Multicopper oxidase
GACN01001724.1	−1.13	1.72	AMP-binding enzyme
GACN01016941.1	−1.27	1.30	Secondary metabolites biosynthesis, transport and catabolism NUDIX domain
GACN01001199.1	−1.57	3.03	2OG-Fe(II) oxygenase superfamily
GACN01025109.1	−1.29	1.75	Anthocyanidin 3-O-glucosyltransferase 4
GACN01017778.1	−1.01	1.74	Secondary metabolites biosynthesis, transport and catabolism Cytochrome P450
GACN01023881.1	−1.38	5.23	PREDICTED: uncharacterized protein
GACN01046804.1	−1.27	2.30	AMP-binding enzyme
GACN01037971.1	−1.09	1.11	Taxadien-5-alpha-ol O-acetyltransferase
GACN01001030.1	−1.48	1.40	Secondary metabolites biosynthesis, transport and catabolism Aromatic amino acid lyase
GACN01045279.1	−1.30	1.44	Probable calcium-binding protein CML45
GACN01027915.1	−1.30	1.96	AMP-binding enzyme C-terminal domain
**0.5D 10D**			
GACN01040109.1	−1.32	−1.45	Probable WRKY transcription factor 70

### GO enrichment analyses of DEGs at 0.5 h and 10 h

Using GO enrichment analysis, the DEGs were divided into three major functional categories named “cellular component”, “molecular function” and “biological process”. We focused on the biological process (GO-BP) category and found that, at 0.5 h, most of the enriched subcategories were related to various signal transduction pathways, and the responses to abiotic and biotic stress. At 10 h, most of enriched subcategories were related to cell size and various secondary metabolite biosynthetic processes. These results indicate that BR-responsive DEGs belong to subcategories relating to petal growth (Table 3).

**Table 3:** DEGs enriched with GO terms.

GO ID	GO name	DEG counts	*p*-value
**H 0.5*vs.****B 0.5*			
GO:0023014	Signal transduction by phosphorylation	4	2.41E−04
GO:0035556	Intracellular signal transduction	6	3.23E−04
GO:0009736	Cytokinin-activated signaling pathway	3	3.18E−03
GO:0009873	Ethylene-activated signaling pathway	5	2.03E−04
GO:0009697	Salicylic acid biosynthetic process	8	3.41E−06
GO:0009738	Abscisic acid-activated signaling pathway	8	2.66E−05
GO:0050832	Defense response to fungus	12	3.61E−08
GO:0006950	Response to stress	11	2.83E−07
GO:0009408	Response to heat	10	1.64E−06
**H 10*vs.*B 0.5**			
GO:0008361	Regulation of cell size	2	1.09E−04
GO:0009932	Cell tip growth	3	1.20E−04
GO:0009664	Plant-type cell wall organization	3	1.23E−04
GO:0008152	Metabolic process	15	2.17E−04
GO:0016099	Monoterpenoid biosynthetic process	3	1.02E−04
GO:0016114	Terpenoid biosynthetic process	3	1.86E−03
GO:0009813	Flavonoid biosynthetic process	3	2.23E−03
GO:0009718	Anthocyanin biosynthetic process	5	9.08E−06

### KEGG pathway enrichment analysis of DEGs

**Figure 4:**
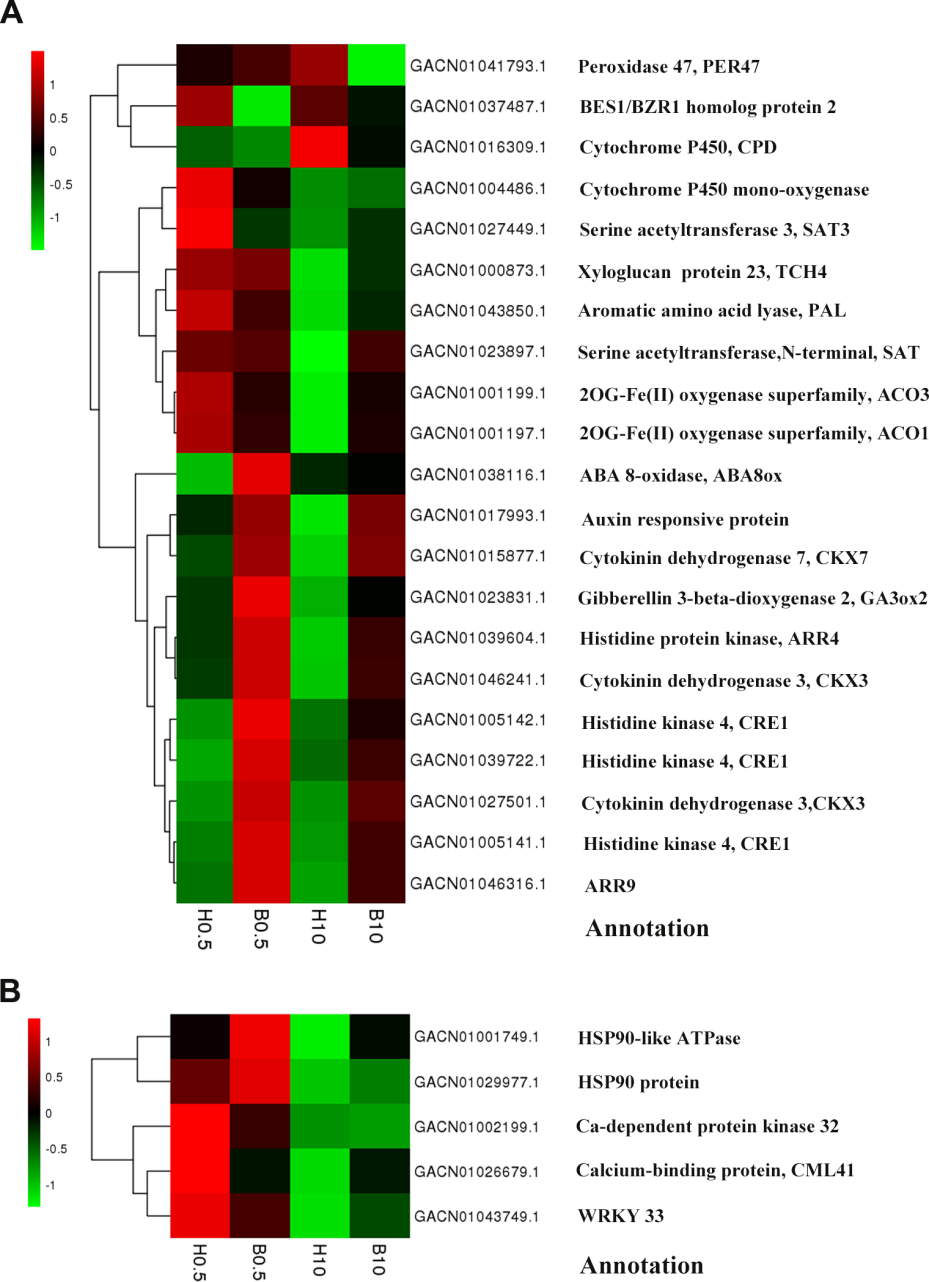
Heat-maps of DEGs involved in “plant hormone signal transduction” and “biotic and abiotic stress”. (A) DEGs involved inplant hormone signal transduction”. (B) DEGs involved in “biotic and abiotic stress”. The bar represents the scale of the expression levels for each gene (log_10_RPKM) in different samples as indicated by green/red rectangles. Green indicates down-regulation of genes, red indicates up-regulation and no change is indicated in black.

We used the KEGG database to determine which pathways were populated by BR-regulated DEGs in petals. In total, 34 DEGs at 0.5 h and 48 DEGs at 10 h were annotated and mapped to various pathways. DEGs involved in plant hormone signal transduction, including the cytokinin, BR and auxin pathways, were significantly enriched comparing the B 0.5 column with H 0.5, and similarly for the 10 h timepoint (Table 4). For example, in the cytokinin pathway, *CRE1* (GACN01005141.1, GACN01005142.1, GACN01039722.1) and two *ARR*genes (GACN01039604.1, GACN01046316.1) were up-regulated at both 0.5 h and 10 h. In addition, one of the most important TFs regulating the BR pathway, a *BES1/BZR1* homolog (GACN01037487.1), was down-regulated at 0.5 h, while at 10 h, an auxin responsive protein (GACN01017993.1) was up-regulated (Fig. 4A). These data show that BRs regulate the expression of genes involved in multiple hormone signaling pathways. Another representative KEGG process was “biotic and abiotic stresses”, where three DEGs (GACN01002199.1, GACN01026679.1, GACN01043749.1) were down-regulated and two DEGs encoded putative HSP90 proteins (GACN01001749.1, GACN01029977.1) were up-regulated at 0.5 h (Fig. 4B). Moreover, we also observed crosstalk between the biosynthetic pathways of multiple hormones, as follows: zeatin biosynthesis, carotenoid biosynthesis, diterpenoid biosynthesis, BR biosynthesis, cysteine and methionine metabolism and phenylalanine metabolism (Table 4). Most DEGs were down-regulated at 0.5 h in these processes, indicating that BRs might regulate multiple hormone biosynthetic pathways.

**Table 4:** KEGG annotations of enriched pathways.

Pathway	ID	H 0.5 *vs.* B 0.5		H 10 *vs.* B 10	
		Count percent (%)	*P*-value	Count percent (%)	*P*-value
Plant hormone signal transduction	ko04075	17.65%	1.08E−03	8.33%	2.85E−03
Biotic and abiotic stress	ko04626	14.71%	5.24E−03	2.08%	8.09E−03
Zeatin biosynthesis	ko00908	8.82%	6.96E−04	4.17%	2.58E−03
Carotenoid biosynthesis	ko00906	2.94%	2.15E−03		
Diterpenoid biosynthesis	ko00904	2.94%	8.28E−03		
Brassinosteroid biosynthesis	ko00905			2.08%	7.07E−03
Cysteine and methionine metabolism	ko00270	5.88%	1.59E−03	6.25%	7.85E−03
Phenylalanine metabolism	ko00360	5.88%	7.66E−03	2.08%	4.81E−03
Phenylpropanoid biosynthesis	ko00940	5.88%	7.49E−03	2.08%	4.77E−03
RNA transport	ko03013	2.94%	8.26E−03	8.33%	2.17E−03
ABC transporters	ko02010			2.08%	1.57E−03

### Genes modulated by BRs in petal growth

**Figure 5:**
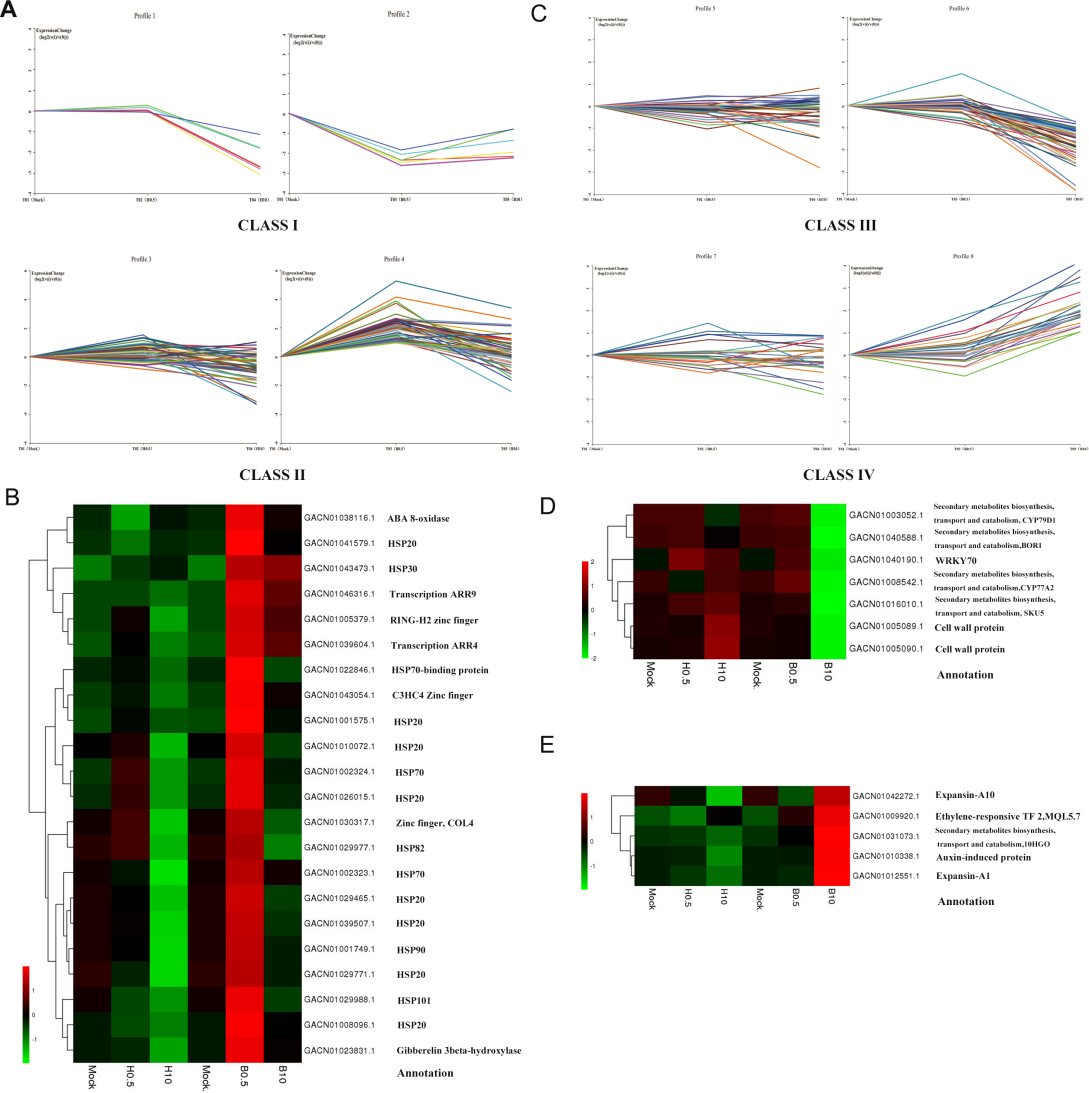
DEGs expression profiles and the heatmaps of some selected DEGs in their profiles. (A) Class I indicated a down-regulated trend during 0–0.5 h of BL treatment and a gradual trend in H_2_O treatment. Class II indicated an up-regulated trend during 0–0.5 h of BL treatment and a gradual trend in H_2_O treatment. (B) Heatmap of DEGs selected from Class II and their annotations. (C) Class III indicated a down-regulated trend during 0.5 h–10 h of BL treatment and a gradual trend in H_2_O treatment. Class IV indicated an up-regulated trend during 0.5 h to10 h of BL treatment and a gradual trend in H_2_O treatment. (D) Heatmap of DEGs selected from Class III and their annotations. (E) Heatmap of DEGs selected from Class IV and their annotations.

In further analysis, we tried to find DEGs mainly involved in the regulation of petal growth by BRs. The DEGs at 0.5 h and 10 h were divided into four classes according to their expression profiles and a hierarchical clustering analysis based on their RPKM (Fig. 5). Class I DEGs were significantly down-regulated after 0.5 h BL treatment and did not change markedly after 0.5 h H_2_O treatment (log_2_ < 1); Class II DEGs were up-regulated after 0.5 h BL treatment and did not change after 0.5 h H_2_O treatment; Class III DEGs weredown-regulated after 10 h BL treatment and did not change after 10 h H_2_O treatment; and Class IV DEGs were up-regulated after 10 h BL treatment and did not change after 10 h H_2_O treatment. As a result, Class I and Class II contained 6 and 48 DEGs, while Class III and Class IV contained 41 and 26 DEGs, respectively. As shown in Fig. 5B, two transcription-related kinases *ARR4* (GACN01039604.1) and *ARR9* (GACN01046316.1) and three zinc finger TFs were discovered in Class II, indicating that, as might be expected, a number of TFs influence petal growth. Fifteen heat shock proteins (HSPs) were found in this class, suggesting that molecular chaperones are important for the regulation of petal growth by BRs at 0.5 h. Interestingly, two genes that regulated putative cell wall proteins are found in Class III, and two expansin genes were identified in Class IV (Fig. 5D). This implies that one aspect of the effect of BRs on petal growth is regulation the expression of cell wall protein and expansin genes in *G. hybrida*.

### Validation of RNA-seq results by qRT-PCR

**Figure 6:**
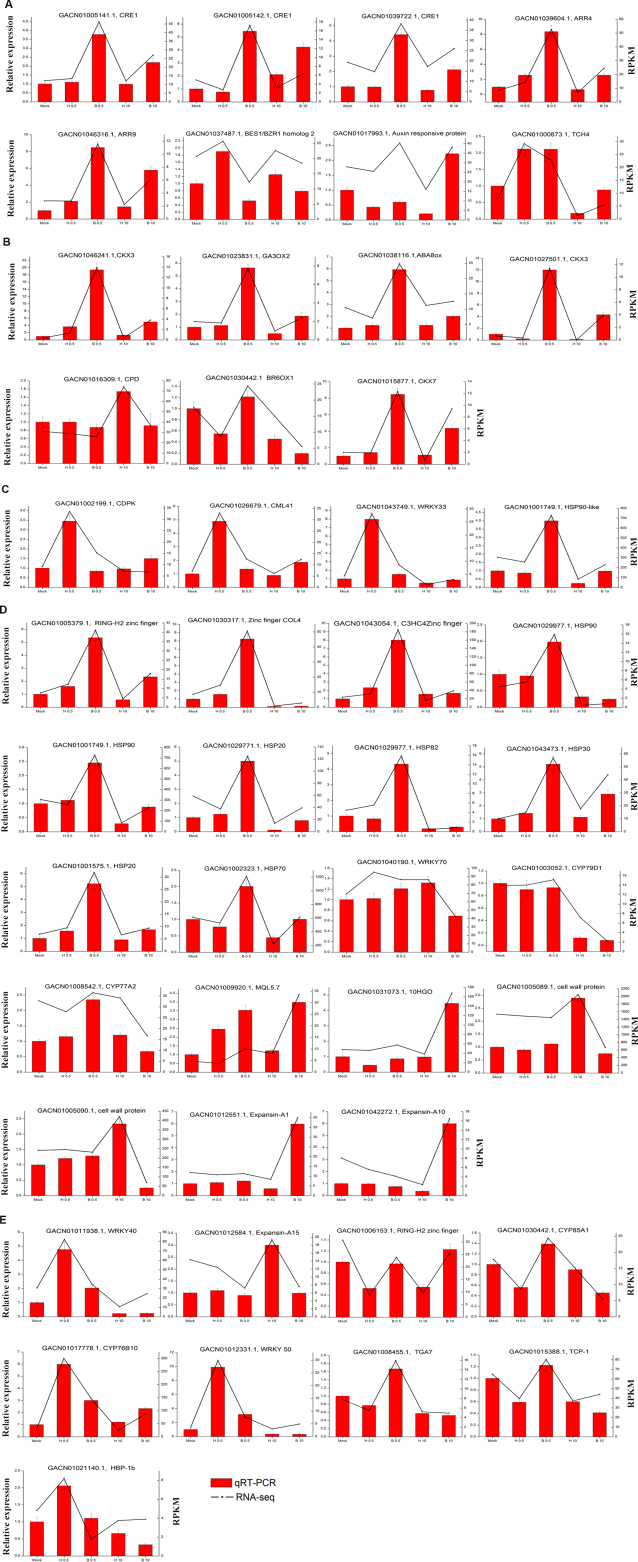
Comparison of unigene expression levels revealed by qRT-PCR and RNA-seq (RPKM). (A) DEGs involved in “Plant hormone signal transduction”. (B) DEGs involved in “hormone biosynthesis”. (C) Four DEGs involved in “plant-pathogen interaction”. (D) DEGs selected from the four classes (nine in Class II, six in Class III and four in Class IV) (E) Nine DEGs selected randomly. *ACTIN* (AJ763915) of *G. hybrida* was used as the normalization control. Three biological repeats were included for each condition.

**Figure 7:**
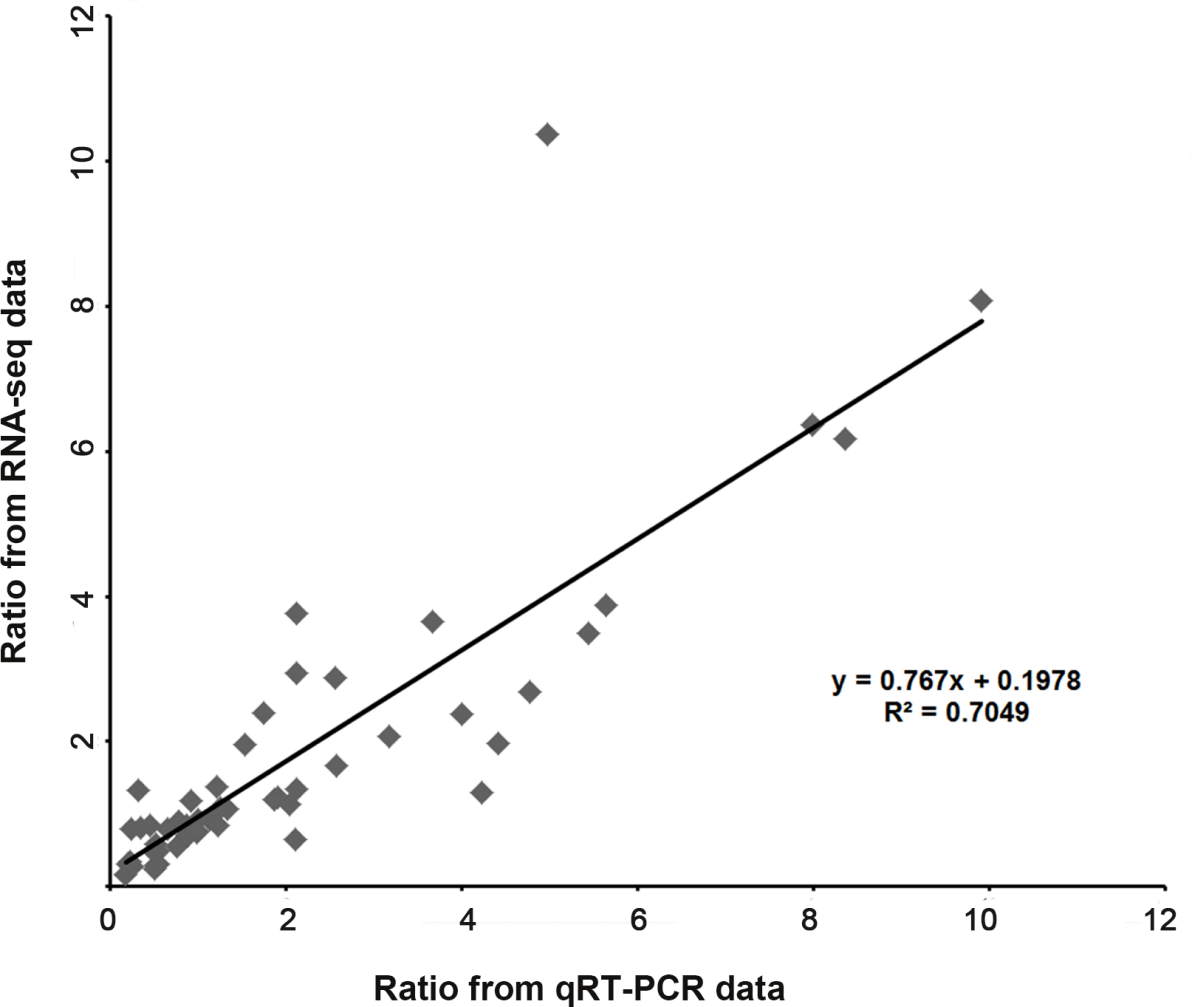
Coefficient analysis of fold change data between qRT-PCR and RNA-seq. Fourteen random unigenes were selected for this analysis. The data of “Mock” was seted as one unit, scatterplots were generated by the ratios of the other four samples from qRT-PCR (*x*-axis) and RNA-seq (*y*-axis).

We validated the expression patterns of several DEGs in the five samples by qRT-PCR. The DEGs chosen were those annotated “plant hormone signal transduction” (Fig. 6A) and “hormone biosynthesis” (Fig. 6B), as well as four genes annotated “biotic and abiotic stresses” (Fig. 6C), nineteen DEGs selected from the four Classes defined above (nine in Class II, six in Class III and four in Class IV ) (Fig. 6D) and nine random DEGs (Fig. 6E). The validation results were consistent with the gene expression patterns identified by RNA-seq. Pearson correlation and linear regression analyses of the fold change of the gene expression ratios between RNA-seq and qRT-PCR showed a significantly positive correlation (Fig. 7). These results highlighted the fidelity and reproducibility of the RNA-seq analysis used in the present study.

## Discussion

### BR modulation of petal growth in *G. hybrida*

Genetic studies suggest that many different pathways act independently to determine flower size. In Arabidopsis, plant hormones, including auxins, cytokinins, BRs and ethylene, play important roles in petal morphogenesis by regulating the expression of key developmental genes (Breuninger & Lenhard, 2010; Weiss, Delgadobenarroch & Egeacortines, 2005). For example, *ARL*, a gene controlling cell expansion during organogenesis, acts downstream of *BRI1* and partially mediates BR-related cell expansion signals during organ growth (Hu, Poh & Chua, 2006). Thus, BRs promote cell expansion to increase floral organ size. Our current research extends these conclusions to *G. hybrida*, in which BR treatment causes petals to elongate significantly (Fig. 1), largely by promoting lengthening of cells in the middle and basal regions (Fig. 2).

Recent studies in Arabidopsis have shown that crosstalk between the BR and GA pathways is brought about by direct interactions between their target transcriptional regulators (Bai et al., 2012; Gallego-Bartolomé & Blázquez, 2012; Li et al., 2012; Oh et al., 2014). In line with this, our previous research showed that GA and BRs act on the same region of the petal when stimulating petal growth in *G. hybrida* (Li et al., 2015). In the present work, therefore, we asked whether these two hormones function coordinately in petal growth. Indeed, we found this to be the case, as the elongation rates of both petals and cells was much higher when they were treated with both hormones as opposed to each hormone independently. We also noted that the speed of response to BR is faster than to GA: the petal elongation rate clearly increased after only 0.5 h BR treatment, but required longer treatment times with GA (Li et al., 2015).

### DEGs regulated by BR involved in cell wall constitution and transcription regulation in *G. hybrida* petals

By comparison of transcriptome sets corresponding to various petal treatments, we identified DEGs that were regulated by BL at early (0.5 h) and late (10 h) timepoints. GO analysis for DEGs enriched in the biological process category showed that subcategories involved in cell size were overrepresented after 10 h BR treatment (Table 3). Further analysis found four genes involved in cell wall constitution in the transcriptome set from the late timepoint. It is well documented that plant cell expansion depends on the activity of various proteins, which loosen cell walls through a complex molecular modification network (Xu et al., 2014). The expansins, which are a group of pH-dependent wall-loosening proteins, are considered to be molecular markers of cell elongation (Bai et al., 2012; Ikeda et al., 2012). We found that two putative cell wall proteins were activated by BR treatment, but two expansin genes were repressed. This suggests that BRs may promote cell elongation by the harmonious expression of a network of genes involved in cell wall constitution after 10 h treatment. The regulation pattern of these genes is consistent with that resulting from GA and ABA treatment (Li et al., 2015).

Transcriptional regulation of key genes is essential for petal growth and a number of TFs controlling petal development have been identified recently (Alvarezbuylla et al., 2010; ÓMaoiléidigh, Graciet & Wellmer, 2014). One of these is *BES1/BZR1*, which plays a central role in BR signaling pathways, as evidenced by the fact that the gain-of-function mutant *bzr1-1D* shows enhanced BR signaling, resulting in increased flower and silique size (Wang et al., 2002; Yin et al., 2002; Yu et al., 2016). Other TFs characterized in Arabidopsis include the TCP gene family, whose members play an important role in organ growth, and the C2H2 zinc finger TFs, such as JAG and RBE, which regulate the transcription of *TCP4* during petal development (Efroni et al., 2008; Jing et al., 2016; Martín-Trillo & Cubas, 2010; Powell & Lenhard, 2012; Schiessl, Muiño & Sablowski, 2014). In *G. hybrida*, we found that several TFs were regulated after only 0.5 h BR treatment, including a homolog of *BES1/BZR1* (GACN01037487.1) and three zinc-finger TFs (GACN01030317.1, GACN01043054.1 and GACN01005379.1), indicating that transcriptional regulation is also important in *gerbera* petal growth.

BR signaling is mediated by HSP90 activity and via trafficking of BIN2-HSP90 complexes into the cytoplasm. In *Arabidopsis*, HSP90, as a molecular chaperone, contributes to BR-mediated gene expression through complex formation with two major TFs, *BZR1* and *BES1* (Samakovli et al., 2014; Shigeta et al., 2015). A recent study reported that HSP90 is pivotal in the transition from vegetative to reproductive phase in Arabidopsis and that depletion of *HSP90* mRNA levels causes extreme variation in the expression of flowering genes, and disturbs flower determination and patterning (Margaritopoulou et al., 2016). We found that a large number of HSP genes were regulated by BR treatment, indicating that HSPs also have a central function in BR-regulated petal growth in *G. hybrida.*

### BRs regulate a wide range of physiological and developmental processes in *G. hybrida*

BRs are involved in a wide range of physiological and developmental processes through their interactions with other phytohormones including auxins, cytokinins, ethylene, GAs, jasmonic acid, ABA, salicylic acid and polyamine in plants (Ahammed et al., 2015; Arteca & Arteca, 2008; Belkhadir & Jaillais, 2015; Wang et al., 2011). Our results in *G. hybrida* support this view, with GO and KEGG analyses implicating BRs in multiple hormone signaling and biosynthesis pathways (Table 4, Fig. 4). In particular, we found that DEGs induced after both 0.5 h and 10 h BR treatment were highly enriched in the cytokinin pathway, which is an evidence for cooperation between BRs and cytokinins in petal growth. In *Arabidopsis*, cytokinins are involved in the regulation of cell division and metabolism, and there is crosstalk with BR in the regulation of plant growth and development (Chi et al., 2016; Hwang, Sheen & Müller, 2012; Saini, Sharma & Pati, 2015; Vercruyssen et al., 2011). In our study, two DEGs (GACN01046316.1, GACN01039604.1), corresponding to the *ARR9* and *ARR4* histidine kinase genes, were clearly up-regulated after 0.5 h BR treatment, indicating that the cytokinin signal pathway might be influenced by BRs in petal growth.

BRs are essential for plant tolerance to environmental factors (drought, flooding, extreme temperatures, salinity) and toxic substances, such as heavy metals, UV-radiation, and ozone (Kim et al., 2012; Kutschera, 2012). BRs may also regulate other phytohormones, such as ABA, SA and ethylene, to induce plant resistance to these abiotic stresses (Ashraf, 2010; Hasan, Hayat & Ahmad, 2011). Our RNA-seq data show that DEGs involved in stress tolerance, including WRKY TFs, were specifically up or down-regulated after 0.5 h or 10 h BL treatment. Together, the above results point to the regulation of petal growth by BRs being a very complex network of processes.

In summary, we report a comprehensive transcriptome dataset relating to petal growth in *G. hybrida* following BR treatment and have identified a group of DEGs that might regulate petal growth. This is a valuable resource for further Asteraceae genomic studies and will also benefit research in other closely related species with high ornamental and economic value. The DEG annotation we carried out should also provide useful candidate genes, especially TFs, for functional analysis of petal growth and may have great significance for the improvement of the ornamental characteristics of *G. hybrida* via molecular breeding.

## Supplemental Information

10.7717/peerj.3382/supp-1 Table S1 List of primers used for gene expression analysis

10.7717/peerj.3382/supp-2 Supplemental Information 2 DEGs after 0.5 h of BL treatment

10.7717/peerj.3382/supp-3 Supplemental Information 3 DEGs after 10 h of BL treatment

10.7717/peerj.3382/supp-4 Supplemental Information 4 Differentially expressed genes in Class I Class I DEGs were significantly down-regulated after 0.5 h BL treatment and did not change markedly after 0.5 h H_2_O treatment (log_2_ < 1).

10.7717/peerj.3382/supp-5 Supplemental Information 5 Differentially expressed genes in Class II Class II DEGs were up-regulated after 0.5 h BL treatment and did not change markedly after 0.5 h H_2_O treatment.

10.7717/peerj.3382/supp-6 Supplemental Information 6 Differentially expressed genes in Class III Class III DEGs were down-regulated after 10 h BL treatment and did not change markedly after 10 h H_2_O treatment.

10.7717/peerj.3382/supp-7 Supplemental Information 7 Differentially expressed genes in Class IV Class IV DEGs were up-regulated after 10 h BL treatment and did not change markedly after 10 h H2O treatment.

## Additional Information and Declarations

### Data Availability

The following information was supplied regarding data availability: The raw data has been supplied as a Supplementary File.
